# Antiphytoviral Activity of Sesquiterpene-Rich Essential Oils from Four Croatian *Teucrium* Species

**DOI:** 10.3390/molecules16098119

**Published:** 2011-09-21

**Authors:** Nada Bezić, Elma Vuko, Valerija Dunkić, Mirko Ruščić, Ivica Blažević, Franko Burčul

**Affiliations:** 1 Department of Biology, Faculty of Science, University of Split, Teslina 12, 21000 Split, Croatia; Email: bezic@pmfst.hr (N.B.); elma@pmfst.hr (E.V.); mrus@pmfst.hr (M.R.); 2 Department of Organic Chemistry, Faculty of Chemistry and Technology, University of Split, Teslina 10, 21000 Split, Croatia; Email: blazevic@ktf-split.hr; 3 Department of Biochemistry, Faculty of Chemistry and Technology, University of Split, Teslina 10, 21000 Split, Croatia; Email: franko@ktf-split.hr

**Keywords:** *β-caryophyllene*, CMV, *Teucrium chamaedrys*, *Teucrium flavum*, *Teucrium montanum*, *Teucrium polium*

## Abstract

The purpose of this study was to compare the essential oil profiles of four Croatian *Teucrium* species (Lamiaceae), as determined by GC and GC/MS, with their antiphytoviral efficiency. A phytochemical analysis showed that *T. polium*, *T. flavum*, *T. montanum* and *T. chamaedrys* are characterized by similar essential oil compositions. The investigated oils are characterized by a high proportion of the sesquiterpene hydrocarbons *β*-caryophyllene (7.1–52.0%) and germacrene D (8.7–17.0%). Other important components were *β*-pinene from *T. montanum* and α-pinene from *T. flavum.* The investigated essential oils were proved to reduce lesion number in the local host *Chenopodium quinoa* Willd. infected with Cucumber Mosaic Virus (CMV), with reductions of 41.4%, 22.9%, 44.3% and 25.7%, respectively.

## 1. Introduction

In the flora of Europe the genus *Teucrium* (Lamiaceae) includes 49 species, of which 13 are widespread in the Croatian flora, including the four species studied in this paper: *T. polium* L., *T. flavum* L., *T. montanum* L. and *T. chamaedrys* L. [1,2,3,4].The essential oils of several other species of the genus *Teucrium*, viz. *T. stocksianum* ssp. *stocksianum* [5], *T. hyrcanicum* [6], *T. maghrebinum* [7], *T. montbtetii* ssp. *heliotropiifolium* [8], *T. scoridum* [9] and *T. salviastrum* [4] have been previously investigated. These oils are characterized by the presence of sesquiterpenes such as caryophyllene, caryophyllene oxide, germacrene D, α-humulene, α-muurolene, (*E)-β-*farnesene and the monoterpene carvacrol. Due to the wide spectrum of biological activities displayed by the essential oils, these compounds are the subject of different researches. The multiple roles of the essential oils and their main components make them natural substances of great importance in several fields such as physiological function of growth, ecological function, development [10], resistance against diseases and insects [11]. They also posses antimicrobial, antiviral, antimycotic, antioxigenic, antiparasitic and insecticidal properties [12,13,14,15,16]. Regarding phytopathogenic viruses, various substances of natural and synthetic origin have been assessed for their antiphytoviral activity [17,18,19,20]. So far, only a few studies have revealed the antiphytoviral activity of pure essential oils [13,17,21,22]. Essential oil of *Satureja montana* L. ssp. *variegata* (Host) P.W. Ball as well as its dominant phenol compounds thymol and carvacrol applied simultaneously with the infecting virus, reduced the number of local lesions on both Tobacco Mosaic Virus (TMV) and Cucumber Mosaic Virus (CMV) infected plants [21]. Essential oil of *Melaleuca alternifolia* (Maiden&Betche) was effective in reducing lesion number in TMV-infected plants [13], while *Plectranthus tenuiflorus* essential oil inhibited Tobacco Necrosis Virus (TNV) infection [17]. Essential oil from Mediterranean endemic plant *Teucrium arduini* L. showed inhibitory effect on the development of infections caused by TMV and CMV [22].

The aim of the study was to determine the volatiles of four Croatian grown *Teucrium* species and evaluate their antiphytoviral effects on the development of local lesions in CMV infected plants.

## 2. Results and Discussion

### 2.1. Essential Oil Composition and Variability

Water distilled essential oils from aerial parts of *T. polium*, *T. flavum*, *T. montanum* and *T. chamaedrys*, collected from different localities (Table 3) have been analysed by GC and GC/MS and 54 different compounds were identified: 33 from *T. polium*, 45 from *T. flavum*, 37 from oil of *T. montanum* and 21 from oil of *T. chamaedrys*, representing 95.3%, 97.3%, 99.5% and 98.3% of the total oil, respectively (Table 1). The yields of the essential oils isolated from plants while in flowering vegetative cycle were: 0.5% from *T. polium*, 0.4% from *T. flavum* and *T. montanum* and 0.3% from *T. chamaedrys.* The main constituents of the investigated essential oils of *Teucrium* species are as follows: in *T. polium β*-caryophyllene (52%) and germacrene D (8.7%); in *T. flavum β*-caryophyllene (23.1%), germacrene D (15.3%) and α-pinene (10.5%); in *T. montanum* germacrene D (17.2%), *β*-pinene (12.3%) and *β*-caryophyllene (7.1%); in *T. chamaedrys β*-caryophyllene (47.6%) and germacrene D (29.0%). Our results thus show that the major compounds in all investigated essential oils were the sesquiterpene hydrocarbons *β*-caryophyllene and/or germacrene D.

Cavaleiro *et al.* [4] reached similar conclusion for endemic species *T. salviastrum* from Portugal. In the oil of *T. polium* ssp. *capitatum* from Crete [8] the most abundant compounds were caryophyllene and the monoterpene carvacrol. Carvacrol was absent in all investigated oils, but in our previous study of *T. arduini* essential oil carvacol was represented in an amount of 1.6% [22]. The composition of *T. polium* from Iran [23] was similar to the investigated oils of *T. flavum* and *T. montanum* regarding the content of α- and *β*-pinene. In the present study we also identified limonene as an important compound of essential oils of *T. polium* (5.9%), *T. flavum* (7.9%) and *T. montanum* (4.6%). These monoterpenes were reported as the main constituents in the oil of *T. chamaedrys* ssp. *chamaedrys* from Iran [6]. In this study, in relatively high percentages, essential oil from *T. flavum* contained α-pinene (10.5%), *β*-pinene (8.4%) and limonene (7.9%), while *T. montanum* essential oil contained *β*-pinene (12.3%) and limonene (4.6%).

### 2.2. Antiphytoviral Activity

A comparison (t-test) of the mean number of lesions on the oil-treated *Chenopodium quinoa* plants with the corresponding control showed that essential oils isolated from the investigated *Teucrium* species significantly reduced CMV infections. The most effective in reducing local lesion number was oil of *T. montanum* (44.3%), followed by *T. polium* (41.4%), *T. chamaedrys* (25.7%) and *T. flavum* (22.9%) (Table 2). The common feature of all investigated oils is the presence of *β*-caryophyllene and germacrene D in relatively high percentages. Our previous investigation had confirmed that *β*-caryophyllene is effective in reducing CMV infection [22]. With the exception of *T. montanum* essential oil, the percentage of *β*-caryophyllene in the oil correlates with antiviral activity of the oil. Essential oil of *T. montanum* showed the strongest antiviral activity, although content of *β*-caryophyllene is lower when compared to the other three oils. The most abundant components from *T. montanum* essential oil, aside from β-caryophyllene, are germacrene D, β-pinene and limonene and as such they can be suggested to be responsible for the better antiphytoviral effect observed. As a comparison, the previously reported essential oil of *Satureja montana* was an inhibitor of TMV and CMV with antiviral activity rates of 29.2% and 24.1%, respectively [21]. Dominant components of this oil were the oxygenated monoterpenes thymol and carvacrol, while the investigated oils from *Teucrium* species are rich in sesquiterpenes. Comparing the percentages of inhibition, essential oils of *T. polium* and *T. montanum* showed significantly stronger antiviral activity against CMV than essential oil of *S. montana* [21]. Other literature data dealing with antiviral activity of essential oils do not compare composition of oils and their antiviral effectiveness. Essential oil of *Melaleuca alternifolia* was previously reported as an inhibitor of TMV [13], while essential oil of *Plectranthus tenuifloru* showed an inhibitory effect against Tobacco Necrosis Virus, Tobacco Mosaic Virus and Tomato Spotted Wilt Virus [17]. Our conclusion is that sesquiterpene-rich essential oils are potent inhibitors of CMV infection and natural substances with possible role in the control of plant virus diseases.

**Table 1:** Phytochemical composition (%) of essential oils of *T. polium*, *T. flavum, T. montanum *and *T. chamaedrys.*

Component	RI VF-5MS	RI CP-Wax 52		*T. polium*	*T. flavum*	*T. montanum*	*T. chamaedrys*	Identification
***Monoterpene hydrocarbons***				**6.3**	**28.4**	**24.4**	**3.9**	
*α*-Pinene	938	-		tr	10.5	1.9	1.0	RI, MS, Co-GC
Camphene	962	-		-	0.1	-	-	RI, MS
*β*-Pinene	982	<1200		0.3	8.4	12.3	1.9	RI, MS, Co-GC
Myrcene	992	<1200		0.1	0.7	4.2	0.2	RI, MS
Limonene	1032	1204		5.9	7.9	4.6	0.6	RI, MS, Co-GC
(*Z*)-*β*-Ocimene	1052	1218		tr	0.6	0.8	0.2	RI, MS
Terpinolene	1089	1286		-	0.2	0.6	-	RI, MS
***Oxygenated monoterpenes***				**12.5**	**3.4**	**12.4**	**0.2**	
Linalool	1099	1548		1.9	1.5	3.6	-	RI, MS, Co-GC
*β*-Thujone	1121	1438		5.7	-	0.3	-	RI, MS, Co-GC
*trans*-Pinocarveol	1147	-		-	0.4	1.2	-	RI, MS
Camphor	1151	1499		1.4	-	1.3	-	RI, MS, Co-GC
Borneol	1176	1719		1.4	-	1.6	-	RI, MS, Co-GC
Terpinen-4-ol	1184	1611		0.2	0.2	1.5	-	RI, MS
Myrtenol	1197	1782		-	0.6	1.2	0.2	RI, MS
*β*-Cyclocitral	1223	1629		-	0.2	-	tr	RI, MS
Linalyl acetate	1252	1553		0.8	0.3	0.5	-	RI, MS, Co-Gc
Bornyl acetate	1285	1570		1.1	0.2	0.2	-	RI, MS, Co-Gc
*α*-Terpenyl acetate	1349	-		-	-	1.0	-	RI, MS
***Sesquiterpene hydrocarbons***				**76.0**	**50.8**	**35.1**	**86.9**	
*α*-Copaene	1377	1484		0.2	0.7	-	0.7	RI, MS
*β*-Bourbonene	1383	1508		0.7	2.6	3.4	3.7	RI, MS
*α*-Gurjunene	1407	1520		-	0.3	-	0.2	RI, MS
*β*-Caryophyllene	1424	1585		52.0	23.1	7.1	47.6	RI, MS, Co-GC
*β* -Copaene	1429	-		1.4	2.7	-	5.7	RI, MS
*trans*-*α*-Bergamotene	1433	-		4.1	-	-	-	RI, MS
(*Z*)-*β*-Farnesene	1454	1639		4.3	2.1	2.9	-	RI, MS
*α*-Humulene	1456	1654		4.6	-	-	-	RI, MS
*allo*-Aromadendrene	1465	1662		-	1.2	-	-	RI, MS
Germacrene D	1481	1692		8.7	15.3	17.2	29.0	RI, MS
*β*-Bisabolene	1494	1729		tr	1.8	1.8	-	RI, MS
*δ*-Cadinene	1517	1745		tr	1.0	2.7	-	RI, MS
***Oxygenated sesquiterpenes***				**tr**	**5.0**	**5.1**	**5.9**	
Spathulenol	1577	2101		tr	1.6	1.9	tr	RI, MS
Caryophyllene oxide	1581	1955		tr	2.6	1.0	4.5	RI, MS, Co-GC
*α*-Cadinol	1655	2208		tr	0.8	2.2	1.4	RI, MS
***Phenolic compounds***				**0.1**	**1.1**	**-**	**0.6**	
*p*-Vinylanisole	1159	-		-	0.5	-	-	RI, MS
Methyl salicylate	1194	-		-	-	-	0.3	RI, MS
*p*-Vinyl-guaiacol	1312	-		-	0.2	-	0.1	RI, MS
Eugenol	1370	2020		0.1	0.4	-	0.2	RI, MS, Co-GC
***Carbonylic compounds***				**tr**	**7.2**	**0.9**	**0.4**	
*n*-Amyl isovalerate	1113	-		-	3.7	0.5	-	RI, MS
3-Octanol acetate	1125	1376		-	0.4	-	-	RI, MS
Isobutyl hexanoate	1155	-		-	0.4	-	-	RI, MS
Butylhexanoate	1193	-		-	0.5	-	-	RI, MS
Hexyl isovalerate	1245	1409		-	0.1	-	-	RI, MS
Isoamyl hexanoate	1256	1457		-	1.6	-	-	RI, MS
6,10,14-Trimethyl-2-pentadecanone	1839	2113		tr	0.5	0.4	0.4	RI, MS
***Hydrocarbons***				**0.4**	**1.4**	**21.6**	**0.4**	
Eicosane	2000	2000		-	-	0.2	-	RI, MS, Co-GC
Heneicosane	2100	2100		tr	-	1.0	0.4	RI, MS, Co-GC
Docosane	2200	2200		-	0.1	1.9	-	RI, MS, Co-GC
Tricosane	2300	2300		tr	0.2	2.8	-	RI, MS, Co-GC
Tetracosane	2400	2400		-	0.1	3.1	-	RI, MS, Co-GC
Pentacosane	2500	2500		0.2	0.3	3.3	-	RI, MS, Co-GC
Hexacosane	2600	2600		-	0.1	3.4	-	RI, MS, Co-GC
Heptacosane	2700	2700		0.1	0.3	2.7	-	RI, MS, Co-GC
Octacosane	2800	2800		0.1	0.1	2.0	-	RI, MS, Co-GC
Nonacosane	2900	2900		tr	0.2	1.2	-	RI, MS, Co-GC
***Total identified (%)***				**95.3**	**97.3**	**99.5**	**98.3**	
***Yield (%)***				**0.5**	**0.4**	**0.4**	**0.3**	

RI-identification by comparison to literature [24] and/or homemade library; MS-identification by NIST02 and Wiley 7 spectral databases; Co-GC- identification confirmed with reference compound; tr‑traces (mean value below 0.1%); - = not identified.

**Table 2:** Effect of *Teucrium flavum*, *T. chamaedris*, *T. polium* and *T. montanum* essential oils on CMV infectivity.

Mean of L.L ± SEM % of inhibition		
Control	7.0 ± 0.5	/
*T. polium*	4.1 ± 0.3 *	41.4
*T. flavum*	5.4 ± 0.3 *	22.9
*T. montanum*	3.9 ± 0.4 *	44.3
*T. chamaedrys*	5.2 ± 0.4 *	25.7

Mean of L.L = the mean number of local lesions; SEM = Standard Error Mean; * Significance reduction in disease compared with control (p ≤ 0.05).

## 3. Experimental

### 3.1. Plant Material

Plant material was collected, as stated in Table 3, in the spring (June) of 2011. Voucher specimens are deposited at the herbarium of the Department of Biology, Faculty of Science, University of Split, Croatia [No.FNSMST 2011: 2, 3, 4 and 5].

**Table 3:** Display of the localities, coordinates, elevations and habitat types of the investigated species of the *Teucrium* genus.

Locality in Croatia	Plant species	Habitat Types	Coordinates: Gauss-Krüger (X,Y)	Altidude a.s.l. (m)
Elevations between Trogir and Prapatnica	*T. polium*	Rocky grassland, once an area affected by fire	X = 5598845Y = 4820965	277
Marjan, hill above town Split	*T. flavum*	Dry grasslands as. *Querco ilici-Pinetum halepensis* Loisel 1971	X = 5614505Y = 4819218	158
Elevations between Trogir and Prapatnica	*T. montanum*	Rocky grassland, once an area affected by fire	X = 5598845Y = 4820965	277
Klis Grlo	*T. chamaedrys*	Rocky grassland in succession towards under-growth and lower forest as. *Carpino orientali-Quercetum virgilianae* Trinajstić 1987	X = 5624263Y = 4826875	356

### 3.2. Isolation of Essential Oils

Aerial parts of plants were dried in a shady place at room temperature for 10 days. Plant tops during flowering were used for the analysis of essential oil composition. Dried aerial parts of plant material (100 g) were subjected to hydrodistillation for 3 h in a Clevenger type apparatus. The obtained essential oil was dried over anhydrous sodium sulphate.

### 3.3. Gas Chromatography and Mass Spectrometry (GC, GC/MS)

Gas chromatography analyses were performed on gas chromatograph (model 3900; Varian Inc., Lake Forest, CA, USA) equipped with flame ionization detector, mass spectrometer (model 2100T; Varian Inc.), non-polar capillary column VF-5MS (30 m × 0.25 mm i.d., coating thickness 0.25 μm) and polar CP Wax 52 (30 m × 0.25 mm i.d., coating thickness 0.25 μm). VF-5MS column temperature was programmed at 60 °C isothermal for 3 min, and then increased to 246 °C at a rate of 3 °C·min^−1^ and held isothermal for 25 min. CP Wax 52 column temperature was programmed at 70 °C isothermal for 5 min, and then increased to 240 °C at a rate of 3 °C·min^−1^ and held isothermal for 25 min. Other chromatographic conditions were: carrier gas helium; flow rate 1 mL·min^−1^; injector temperature 250 °C; volume injected 1 μL; split ratio 1:20; FID detector temperature 300 °C. MS conditions: ionization voltage 70 eV; ion source temperature 200 °C; mass scan range: 40–350 mass units.

### 3.4. Data Analysis and Data Evaluation

The individual peaks were identified by comparison of their retention indices (relative to C_8_-C_40_ *n*-alkanes for VF-5MS and CP Wax 52) to those from a homemade library, literature [24] and/or authentic samples, as well as by comparing their mass spectra with literature [24], Wiley 7 MS (Wiley, New York, NY, USA) and NIST02 (Gaithersburg, MD, USA) mass spectral databases. The homemade library was created from authentic compounds obtained commercially and from the main components of many essential oils obtained during our previous studies. The component percentages were calculated as mean values form the GC and GC-MS peak areas using the normalization method (without correction factors).

### 3.5. Plant Host for Antiphytoviral Studies

Seeds of *Chenopodium quinoa* Willd. were sown in trays containing Klasmann universal compost and maintained in a growth chamber (26 °C, 16:8 h light/dark cycle) with watering as required. When the seedlings were large enough to handle they were transplanted individually into perforated styrofoam which contained fresh compost for ten days and then into 10 cm plastic pots with fresh compost. Plants were grown in a growth chamber under same conditions. Experimental plants were selected three to four weeks after sowing, when they had eight true leaves. Care was taken to ensure that the experimental plants were as uniform in size as possible.

### 3.6. Solution of Essential Oil

Spray solution containing 500 ppm of essential oil in distilled water was prepared for testing the antiphytoviral effect. To overcome insolubility of the oil in water 0.05 mL of Tween 80 was mixed with 25 µL of the oil and 50 mL of distilled water. Prepared solution was immediately sprayed to the test plants. 

### 3.7. Viral Inoculums

Viral inoculum was prepared from leaf tissue obtained from *Nicotiana megalosiphon* Van Heurck & Muell. Arg. plants previously infected with Cucumber Mosaic Virus (CMV). Leaves of systemically infected leaf material (3-5) were ground with 10 mL of inoculation buffer (0.06 mol/L phosphate buffer, pH = 7.0) in a mortar. Sap extract was diluted with the same inoculation buffer to give a suitable number of discrete local lesions on test plants. The inoculum prepared in this way was used to inoculate local host *Chenopodium quinoa*.

### 3.8. Application to the Local Host Plants

Spray solution of essential oil was applied to *Chenopodium quinoa* plants for two successive days prior to CMV inoculation. Control spray was of distilled water and Tween 80. Plants were sprayed with either an oil or control solution. The second day of treatment plants were left to dry for 20 min and then inoculated with prepared viral inoculum. All treatments were repeated for three times on plants selected for uniformity and grown in a growth chamber (26 °C; 16:8 h light/dark cycle). Local lesions were counted 6th day post inoculation and the inhibition percentage was calculated by comparing the number of viral lesions on treated and control group according to the formula:

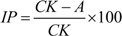

where IP = antiviral inhibition in %, CK = average number of viral lesions on the control group; A = average number of viral lesions on the essential oil treated group.

### 3.9. Statistical Analysis

The significance of difference between mean value for treatment and control was estimatedstatistically using one tailed Student t-test (GraphPad InStat software).

## 4. Conclusion

Our previous investigations as well as the investigation carried out in this study confirmed that essential oils are potent antiphytoviral agents. Based on the limited number of publications published so far in this field, it could be concluded that there is no universal mode of antiphytoviral action of essential oils. *β*-Caryophyllene, the main component of the investigated *Teucrium* oils, and known to be effective antiphytoviral agent, is responsible for the observed antiviral activity. However it is also possible that some other components, which may be present in very small quantities, contribute to its effectiveness. Some of the oil components are already known as antiphytoviral agents, but many others must be tested to answer the question about the antiphytoviral mechanism of essential oils.
